# Supplementary material for: Microfluidic Affinity Selection of B-Lineage Cells from Peripheral Blood for Minimal Residual Disease Monitoring in Pediatric B-Type Acute Lymphoblastic Leukemia Patients
Source: Int J Mol Sci. 2024 Oct 2;25(19):10619. doi: 10.3390/ijms251910619 (PMC11477226; doi:10.3390/ijms251910619)
Supplement: Supplementary file 1 [file ijms-25-10619-s001.zip › ijms-3221544-supplementary.pdf]

International Journal of *Molecular Sciences*

MDPI

Supporting Information

Microfluidic Affinity Selection of B-lineage Cells from Peripheral Blood for Minimal Residual Disease Monitoring in Pediatric B-type Acute Lymphoblastic Leukemia Patients

Małgorzata A. Witek [1,2], Nicholas E. Larkey [2,3], Alena Bartakova [4], Mateusz L. Hupert [4], Shalee Mog [2], Jami K. Cronin [5], Judy Vun [5], Keith J. August [5]* and Steven A. Soper [1,2,3,4,6,7]*

1 Department of Chemistry, The University of Kansas, Lawrence, KS 66047, USA
2 Center of BioModular Multiscale Systems for Precision Medicine, Lawrence, KS 66045, USA
3 Department of Cancer Biology, The University of Kansas Medical Center, Kansas City, KS 66160, USA
4 Biofluidica Inc., San Diego, CA 92121, USA
5 Hematology/Oncology/BMT, Children's Mercy Kansas City, Kansas City, MO 64108, USA
6 Bioengineering Program, The University of Kansas, Lawrence, KS 66045, USA
7 Department of Mechanical Engineering, The University of Kansas, Lawrence, KS 66045, USA
* Correspondence: kjaugust@cmh.edu (K.A.); ssoper@ku.edu (S.-A.S.)

Monoclonal antibody (mAb) labeling with sulfosuccinimidyl-4-(N-maleimidomethyl)cyclohexane-1- carboxylate (Sulfo-SMCC). mAb labeling involved addition of 6 µL (50x excess) of maleimidecrosslinker sulfo-SMCC (10 mg/mL in nuclease free water) to 0.5 mg mAb in 500 µL of water followed by incubation for 1.5 h at room temperature on a rocker. Following reaction, the mAb was purified using a Zeba column (with exchanged buffer for PBS pH 7.4) to remove excess non-reacted sulfo-SMCC. mAbSMCC in PBS pH 7.4 was stored up to 3 d at 4°C for cell selection device modification. When non-lyophilized mAbs were used, which contained sodium azide, the mAb was purified using a Zeba column prior to SMCC labeling.

Modification of the cell selection chip with oligonucleotide linkers. A UV/O_3-activated device was flooded with a solution of 20 mg/mL EDC and 2 mg/mL NHS in 0.1M MES (adjusted to pH 4.8) and incubated at room temperature. After 20 min, vacuum pump was used to remove solution from the chip and immediately after that, a 40 µM solution of the amine- modified ssDNA linker in PBS buffer (pH 7.4) was introduced into the device using syringe and incubated for at least 2 h at room temperature or overnight at 4°C. After the reaction was complete, the microfluidic chip was rinsed with 500 µL PBS (pH 7.4) at 40 µL/min and 0.3 M DTT in carbonate buffer (pH 9) for 30 min to reduce the 3'-disulfide group into a reactive sulfhydryl moiety (-S-H). The microfluidic chip was rinsed with 100 µL PBS (pH 7.4) at 200 µL/min, and immediately an aliquot of the modified mAb-SMCC was introduced (0.5 mg/mL). The reaction proceeded for at least 2 h on ice or overnight at 4°C.

Flow cytometry

Flow cytometry (FC) was performed with a BD Accuri C6 plus (BD Biosciences, Piscataway, NJ) equipped with a blue (488 nm, 20 mW) and red laser (640 nm, 12.5 mW), two light scatter detectors, and four fluorescence detectors with optical filters: FL1 533/30 nm, FL2 585/40 nm, FL3 >670 nm, and FL4 675/25 nm optimized for the detection of FITC, PE, PerCP-Cy™5.5, and APC. Ten thousand events for the selected gated region were counted for cells prepared from healthy donor's blood. Ten thousand events were collected for all the experiments with cell lines and 1000 events for standard beads. Data acquisition and analysis was performed using BD CSampler Plus software. Prior to staining, the cell samples were blocked with human IgG and incubated at 4ºC. The following samples for healthy donor mononuclear cells (isolated with Histopaque-1077, Sigma) and SUP-B15

were prepared for analysis: (i) Unstained cells serving as an autofluorescence control; (ii) cells stained with 10 µL of 0.1 mg/mL isotype control, IgG_1-APC mAbs (R&D Systems, Minneapolis, MN); (iii) cells incubated with 10 µL of 0.1 mg/mL anti-human CD19 -APC. (iv) Standard beads (Quantum™ Simply Cellular® (QSC) anti Mouse IgG (Bangs Lab.)) incubated with mouse anti-human antibodies used in point (iii). Standard beads were used to create a calibration curve used for evaluation of the level of CD19 receptors. While staining, cells and beads were incubated in the dark at 4º C for 30 min. Beads were used for the quantitative analysis of cellular antigen expression. When stained with the same antibody that is used to label cells, they permit determination of the Antibody Binding Capacity (ABC) of the cells. After staining, cells and beads were washed three times with 1 mL of cold PBS/0.5% BSA. When analyzed in FC, beads displayed 5 populations with the receptor number in the range between 0 and 529,000. Cells and QSC beads were run on the same day with the same instrument settings.

FC at the clinical testing laboratory (Children's Mercy Hospital, CMH)

A sample of bone marrow was stained for CD9, CD10, CD13, CD19, CD20, CD22, CD34, CD38, CD58, CD45. The leukemic cells abnormally express CD19 (bright), CD10 (bright), CD58 (bright), CD20 (dim to negative), CD34 (mostly negative), with normal expression of CD9, CD38, CD45, Syto16 without CD13. This finding is consistent with minimal residual B lymphoblastic leukemia/lymphoma. For example, precursor B-cell acute lymphoblastic leukemia blasts are identified by the expressions of CD19, CD10, CD38 (dim), CD22 (dim), CD9, CD58, CD34 (small subset, 15%), CD45 (dim to negative), and no expression of CD20 or CD13. Other samples can identify leukemic blasts based on cell being positive for CD19, CD10 (bright), CD22, CD34 (dim and partial, 50%), TdT positive, CD38 (dim), HLA-DR, CD58, CD123 (dim), CD33 (partial, 30%), CD45 (dim to negative); they are negative for CD20, kappa, lambda, CD9, CD71, CD56, T-cell and other myelomonocytic markers. The finding is consistent with precursor B-cell acute lymphoblastic leukemia.

Samples at CMH were processed and analyzed for MRD using LSR Fortessa X-20 with 4 lasers and 15 detectors (BD Biosciences). The overall sensitivity of the assay is estimated as 0.01% but may vary based on the blast immunophenotype and sample quality. The leukemic cells present <0.01% with respect to nucleated mononuclear cells, are at level below enumeration for this assay and of uncertain clinical significance, and for the COG reporting purposes the result is 0% MRD.[1]

Fluorescence In Situ Hybridization (FISH) and multiparameter FC (MFC) assay performed at CMH have not been approved by the U.S. Food and Drug Administration. The performance characteristics of all immunohistochemical stains and/or flow cytometric tests were determined as part of an ongoing quality assurance program and in compliance with federally mandated regulations drawn from the Clinical Laboratory Improvement Act of 1988 (CLIA '88).

Table S1. Protocol for cell staining on the autostainer.

Step/Procedure	Volume/Time
Cells deposited on a glass slide from 1xPBS	200 µL
Fixation, 3% PFA	100 µL, 15 min.
Wash, 1x PBS/0.005% Tween	100 µL
Dry, Air	Air blow
Staining (CD10, CD34 in 1xPBS)	100 µL, 40 min.
Wash, 1x PBS/0.005% Tween	2x100 µL
Poration, 0.1% Triton X-100 in water	100 µL
Wash, 1x PBS/0.005% Tween	100 µL
Staining (TdT, DAPI in water)	100 µL, 40 min.

Wash, 1x PBS/0.005% Tween	3x100 µL
Deposit 1xPBS for cover plate seal	100 µL

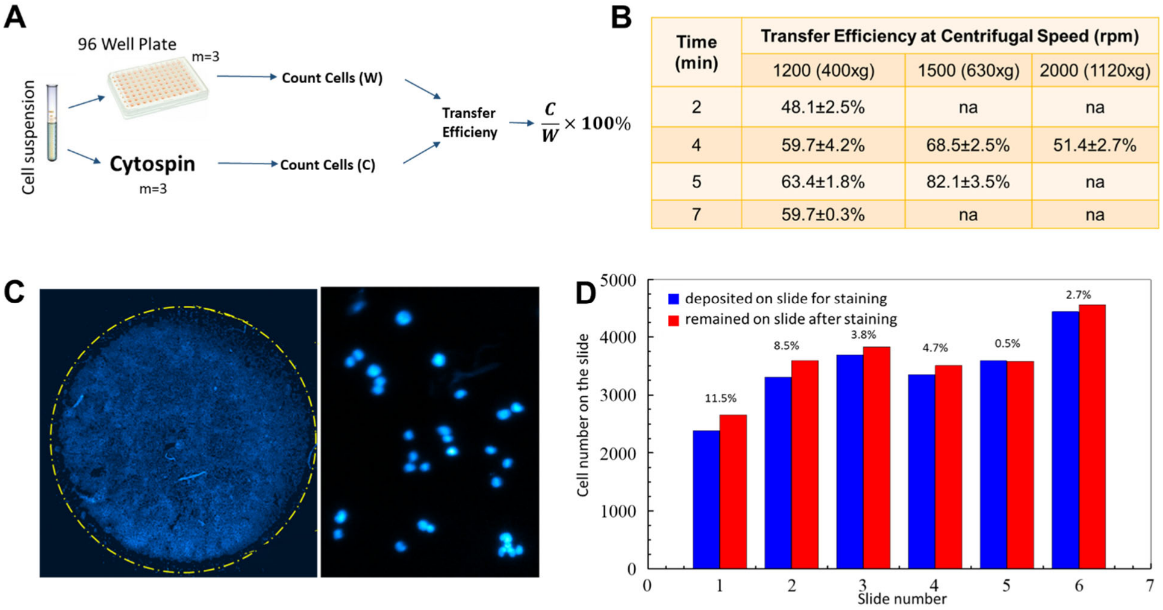

Figure S1. (**A**) Evaluation of the cytospin protocol for cell transfer. (**B**) Results for the cytospin efficiency at different centrifugal forces, na – not assessed. (**C**) Stitched image and a closeup of 32 SUP-B15 cells deposited on a microscope slide and stained with DAPI after fixation to the glass surface. (**D**) Difference in cell number enumerated by the automated Image J script after depositing and attaching to the slide and after washing steps.

Immunophenotyping of B-ALL.

B-ALL is a malignant clonal proliferation of lymphocytes expressing an immature phenotype. B- ALL are classified according to the stage of differentiation in the bone marrow. They are classified as: pro-B, common and pre-B. [2] Table S4 presents phenotypes that were evaluated in the study and their comparison to the presence on normal mature B- cells and type 4 hematogones found in blood. [3-5]

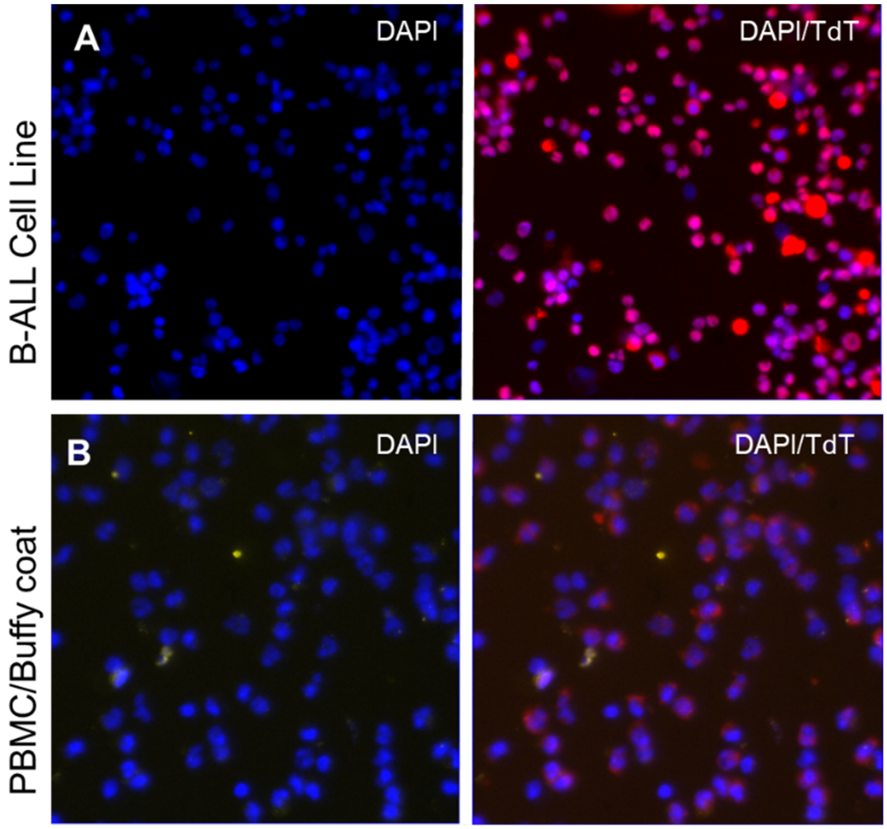

Figure

Figure S2. Different TdT staining patterns in B-ALL model cell line, SUP-B15 (**A**) and normal PBMCs (**B**), isolated from healthy donor samples. Cells in panel A are considered TdT(+) while in panel B show TdT(-) phenotype.

Table S2. Phenotypes of B lineage ALL cancer cells [3-5].

B-cell ALL staging detected in Blood	CD19	Nuclear TdT	CD10	CD34
Early B-pro ALL	+	+	-	+
Pre-B-ALL	+	+	+	+
Common B-ALL	+	+	+	
Mature B-cell ALL (Burkitt leukemia)	+	-	+/-	+/-
Normal mature B-cell	+	-	-	+/-
Normal mature precursor B cells (i.e., mature hematogones/resting B cells in blood)	+	-	+/-	-

In B-ALL patients, when evaluating BMA for presence of B-ALL cells, distinguishing hematogones from leukemic cells may be difficult because hematogones and lymphoblasts often show morphologic and immunophenotypic similarities.[3] During normal B-cell development, hematogones appear in BMA are immature with CD19(+)/CD10(+)/CD34(+)/TdT(+) phenotype, however, hematogones found in blood are mature (resting B-cells) with CD19(+)/CD10(+)/(-)/CD34(-)/TdT(-) phenotype subsets [3].

Table S3. Disease characteristic for pediatric patients diagnosed with B-ALL.

Patient Study ID	Age (yo)	Sex (M/F)	t(9;22)	Double trisomies (4,10)	KMT2A rearrangement	t(12;21)	iAMP21	CNS Status	Induction Chemotherapy	Consolidation Chemotherapy
Pt. 1	3	Female	No	No	No	No	No	CNS1	High Risk	High Risk
Pt. 2	2	Male	No	No	No	**Yes**	No	CNS1	Standard Risk	Standard Risk
Pt. 3	3	Male	No	**Yes**	No	No	No	CNS1	Standard Risk	Standard Risk
Pt. 4	3	Female	No	No	No	**Yes**	No	CNS1	Standard Risk	Standard Risk
Pt. 5	2	Male	No	No	No	**Yes**	No	CNS1	Other	Standard Risk
Pt. 6	2	Male	No	No	No	No	No	CNS1	Standard Risk	High Risk
Pt. 7	2	Female	No	No	No	No	No	CNS1	Standard Risk	High Risk
Pt. 8	4	Male	No	**Yes**	No	No	No	CNS1	Standard Risk	Standard Risk
Pt. 9	2	Male	No	No	No	**Yes**	No	CNS1	High Risk	High Risk
Pt. 10	3	Male	No	No	No	**Yes**	No	CNS1	High Risk	High Risk
Pt. 11	11	Male	No	No	No	No	No	CNS1	High Risk	High Risk
Pt. 12	4	Female	No	**Yes**	No	No	No	CNS1	Standard Risk	Standard Risk
Pt. 14	4	Male	No	No	No	No	No	CNS1	Standard Risk	High Risk
Pt. 15	11	Female	No	**Yes**	No	No	No	CNS1	High Risk	High Risk
Pt. 16	8	Female	No	**Yes**	No	No	No	CNS1	Standard Risk	Standard Risk
Pt. 17	3	Female	No	No	No	No	No	CNS1	Standard Risk	Standard Risk
Pt. 18	6	Male	No	No	No	No	No	CNS1	Standard Risk	Standard Risk
Pt. 19	3	Female	No	**Yes**	No	No	No	CNS1	Standard Risk	Standard Risk
Pt. 20	5	Male	No	No	No	No	No	CNS1	Standard Risk	High Risk
Pt. 21	10	Female	No	**Yes**	No	No	No	CNS1	High Risk	High Risk

Note: CNS1 status: <5 WBC/µL in CSF and no blasts detected. Risk stratification algorithms use clinically relevant risk factors such as white blood cell count (e.g., < vs. ≥50 × 10^6/mL) and minimal residual disease (e.g., < vs. ≥0.01%; MRD). The Children's Oncology Group B-ALL algorithm includes National Cancer Institute (NCI) RG, clinical variables, sentinel favorable and unfavorable risk genetics, flow cytometric MRD of peripheral blood on induction day 8, and BMA on induction day 29 and at end of consolidation [6].

Table S4. B-cell counts isolated in healthy donors.

Healthy Donor ID	DAPI(+)/CD19(+)/mL	DAPI(+)/CD19+/TdT(+)/mL
H1	1429	1.5
H2	1432	10
H3	592	11
H4	2570	10.5
H5	2558	18
H6	1322	4.5
H7	1372	6

Table S5. B-cells and B-ALL counts isolated during microfluidics MRD assessment.

Pt ID	B-cells (CD19+) and CLC (CD19+/TdT+) counts per mL detected in blood of B-ALL pediatric patients											
	Induction therapy						Consolidation therapy					
	Day 8		Day 15		Day 22		Day 29		Day 57		Day 85	
	CD19+	CD19+ and TdT+	CD19+	CD19+ and TdT+	CD19+	CD19+ and TdT+	CD19+	CD19+ and TdT+	CD19+	CD19+ and TdT+	CD19+	CD19+ and TdT+
**Standard Risk Patients**												
002	422	**29**	377	**5**	770	**124**	2167	**723**	562	**38**	422	**24**
003	934	**116**	2995	**161**	4172	**179**	15655	**462**	673	**79**	298	**18**
004	3302	**65**	8024	**294**	14,042	**93**	8422	**71**	582	**68**	205	**155**
005	3862	**59**	6054	**79**	4163	**74**	4405	**172**	171	**23**	15	**9**
008	2091	**28**	1826	**171**	877	**5**	1983	**48**	711	**39**	908	**97**
012	1908	**8**	2822	**65**	3179	**35**	3276	**48**	445	**97**	438	**7**
014	249	**8**	1160	**27**	3172	**117**	956	**8**	776	**498**	8	**3**
016	1670	**526**	11601	**329**	14	**4**	24	**12**	43	**19**	35	**16**
017	18	**9**	-	**-**	30	**10**	33	**10**	38	**12**	118	**16**
018	81	**14**	22	**5**	28	**12**	-	**-**	29	**19**	76	**39**
019	67	**37**	-	**-**	-	-	42	**18**	66	**19**	71	**51**
**Avg**	**1328**	**82**	**3876**	**126**	**3045**	**65**	**3696**	**157**	**372**	**83**	**236**	**40**
**Median**	**934**	**29**	**2822**	**79**	**2025**	**55**	**2075**	**48**	**445**	**38**	**118**	**18**
**High Risk Patients**												
001	1298	**228**	1917	**519**	727	**6**	3146	**565**	371	**104**	158	**55**
006	15852	**210**	5320	**201**	4206	**94**	1578	**134**	101	**27**	49	**4**
007	11372	**469**	9601	**779**	7265	**61**	9344	**1044**	33	**9**	59	**29**
009	2484	**173**	2058	**20**	725	**114**	445	**11**	124	**63**	1761	**420**
010	1221	**72**	2458	**41**	408	**181**	1200	**90**	1987	**754**	**Patient died**	
011	2671	**7**	2809	**680**	2925	**18**	860	**24**	1591	**857**	65	**25**
015	21	**3**	9	**4**	27	**2**	35	**3**	2	**1**	24	**16**
020	32	**6**	20	**5**	14	**10**	14	**3**	40	**23**	7	**3**
021	21	**8**	183	**36**	120	**25**	9	**3**	319	**144**	8	**5**
Avg.	**3886**	**131**	**2708**	**254**	**1824**	**57**	**1848**	**209**	**508**	**220**	**266**	**70**
Median	**1298**	**72**	**2058**	**41**	**725**	**25**	**860**	**24**	**124**	**63**	**54**	**21**

Note: CD19(+) refers to normal B-cells, CD19(+)/TdT(+) refers to B-ALL circulating leukemia cell (CLC).

Table S6. WBC and CLC's count during the induction (day 8 – day 28) and consolidation therapy (day 29 - day 85). Red cells in the table mark MRD(+). * - Red cells: MRD (+), blue cells: MRD(-). Microfluidic MRD(+) defined as >25 CLCs/mL, **- MRD(+) > $5x10^{-4}$ % with respect to WBC in the third column. ***- standard of care MFC MRD (≥0.01%) tested from peripheral blood (PB, day 8) and bone marrow aspirate (BMA, day 29 or day 85).

Pt. ID	Time of test (day)	WBC /mL	CLCs/1 mL MRD (+)*	Microfluidic MRD (+) **	MFC/PB or MFC/BMA***	Disease complete remission as of 03.26.20	Disease complete remission as of 07.18.24
**1**	8	1.26E+06	114	9.05E-03	0.01		
	15	1.33E+06	259.5	1.95E-02	Not tested		

	22	2.68E+06	3	1.12E-04	Not tested		
	29	8.53E+06	282.5	3.31E-03	0		
	57	1.69E+06	52	3.08E-03	Not tested		
	85	4.91E+06	27.5	5.60E-04	Not tested	yes	Relocated; yes, at last visit: 1/6/2022
2	8	4.48E+06	14.5	3.24E-04	0		
	15	2.83E+06	2.5	8.83E-05	Not tested		
	22	4.50E+06	62	1.38E-03	Not tested		
	29	3.72E+06	361.5	9.72E-03	0		
	57	4.47E+06	19	4.25E-04	Not tested		
	85	1.79E+06	12	6.70E-04	Not tested	yes	LTFU; yes, at last visit 7/28/2022
3	8	1.98E+06	58	2.93E-03	0.008		
	15	2.11E+06	80.5	3.82E-03	Not tested		
	22	5.73E+06	89.5	1.56E-03	Not tested		
	29	1.27E+07	231	1.82E-03	0		
	57	2.68E+06	39.5	1.47E-03	Not tested		
	85	4.73E+06	9	1.90E-04	Not tested	yes	yes
4	8	2.51E+06	32.5	1.29E-03	0.04		
	15	1.63E+06	147	9.02E-03	Not tested		
	22	4.39E+06	46.5	1.06E-03	Not tested		
	29	7.04E+06	35.5	5.04E-04	0		
	57	5.62E+06	34	6.05E-04	Not tested		
	85	4.53E+06	77.5	1.71E-03	Not tested	na (relocated)	na (relocated)
5	8	1.58E+06	29.5	1.87E-03	0		
	15	7.50E+05	39.5	5.27E-03	Not tested		
	22	1.57E+06	37	2.36E-03	Not tested		
	29	2.16E+06	86	3.98E-03	0		
	57	2.45E+06	11.5	4.69E-04	Not tested		
	85	2.89E+06	4.5	1.56E-04	0	yes	yes
6	8	5.32E+06	105	1.97E-03	1.8		
	15	8.30E+05	100.5	1.21E-02	Not tested		
	22	2.22E+06	47	2.12E-03	Not tested		
	29	3.64E+06	67	1.84E-03	0		

	57	5.60E+05	13.5	2.41E-03	Not tested		
	85	8.10E+05	2	2.47E-04	Not tested	yes	yes
**7**	8	2.13E+06	234.5	1.10E-02	1.8		
	15	2.24E+06	389.5	1.74E-02	Not tested		
	22	4.56E+06	30.5	6.69E-04	Not tested		
	29	1.05E+07	522	5.00E-03	**0.005**		
	57	4.80E+05	4.5	9.38E-04	Not tested		
	85	2.80E+05	14.5	5.18E-03	Not tested	yes	yes
**8**	8	8.00E+05	14	1.75E-03	0.3		
	15	2.56E+06	85.5	3.34E-03	Not tested		
	22	1.95E+06	2.5	1.28E-04	Not tested		
	29	4.34E+06	24	5.53E-04	0.004		
	57	2.74E+06	19.5	7.12E-04	Not tested		
	85	4.47E+06	48.5	1.09E-03	0	yes	yes
**9**	8	7.50E+05	86.5	1.15E-02	0		
	15	6.10E+05	10	1.64E-03	Not tested		
	22	6.40E+05	57	8.91E-03	Not tested		
	29	1.10E+06	5.5	5.00E-04	0.2		
	57	8.80E+05	31.5	3.58E-03	Not tested		
	85	2.40E+05	210	8.75E-02	0	yes	yes
**10**	8	6.20E+05	36	5.81E-03	0.03		
	15	5.70E+05	20.5	3.60E-03	Not tested		
	22	1.49E+06	90.5	6.07E-03	Not tested		
	29	1.58E+07	45	2.85E-04	0		
	57	1.48E+06	377	2.55E-02	Not tested	died	died
**11**	8	1.12E+06	3.5	3.13E-04	0.015		
	15	1.14E+06	340	2.98E-02	Not tested		
	22	1.69E+06	9	5.33E-04	Not tested		
	29	2.40E+06	12	5.00E-04	0		
	57	9.60E+05	428.5	4.46E-02	Not tested		
	85	1.11E+06	12.5	1.13E-03	Not tested	yes	yes
**12**	8	6.10E+05	4	6.56E-04	0.68		
	15	5.60E+05	32.5	5.80E-03	Not tested		
	22	1.12E+06	17.5	1.56E-03	Not tested		
	29	5.39E+06	24	4.45E-04	0		

	57	2.61E+06	48.5	1.86E-03	Not tested		
	85	2.58E+06	3.5	1.36E-04	Not tested	yes	yes
**14**	8	3.80E+05	4	1.05E-03	0		
	15	4.10E+05	13.5	3.29E-03	Not tested		
	22	4.04E+06	58.5	1.45E-03	Not tested		
	29	4.33E+06	4	9.24E-05	0		
	57	2.87E+06	249	8.68E-03	Not tested		
	85	3.28E+06	1.5	4.57E-05	Not tested	yes	yes
**15**	8	6.00E+05	1.5	2.50E-04	0.01		
	15	1.90E+05	2	1.05E-03	Not tested		
	22	2.70E+05	1	3.70E-04	Not tested		
	29	1.06E+06	1.5	1.42E-04	0.12		
	57	7.13E+06	0.5	7.01E-06	Not tested		
	85	2.66E+06	8	3.01E-04	Not tested	yes	yes
**16**	8	3.93E+06	263	6.69E-03	0		
	15	3.44E+06	164.5	4.78E-03	Not tested		
	22	5.43E+06	2	3.68E-05	Not tested		
	29	1.50E+07	6	4.01E-05	0		
	57	4.58E+06	9.5	2.07E-04	Not tested		
	85	7.20E+06	8	1.11E-04	Not tested	na (relocated)	na (relocated)
**17**	8	8.04E+06	4.5	5.60E-05	0.24		
	15	3.17E+06	na	na	Not tested		
	22	3.53E+06	5	1.42E-04	Not tested		
	29	9.94E+06	5	5.03E-05	0		
	57	6.22E+06	6	9.65E-05	Not tested		
	85	7.25E+06	8	1.10E-04	Not tested	yes	yes
**18**	8	3.10E+06	7	2.26E-04	0		
	15	5.34E+06	2.5	4.68E-05	Not tested		
	22	6.33E+06	6	9.48E-05	Not tested		
	29	Not tested	Not tested	Not tested	Not tested		
	57	5.07E+06	9.5	1.87E-04	Not tested		
	85	3.95E+06	19.5	4.94E-04	Not tested	yes	yes
**19**	8	5.23E+06	18.5	3.54E-04	0.004		
	29	6.20E+06	9	1.45E-04	0		

	57	9.59E+06	9.5	9.91E-05	Not tested		
	85	1.26E+07	25.5	2.03E-04	Not tested	yes	yes
**20**	8	1.78E+06	3	1.69E-04	1.77		
	15	1.28E+06	2.5	1.95E-04	Not tested		
	22	7.01E+06	5	7.13E-05	Not tested		
	29	9.86E+06	1.5	1.52E-05	0.011		
	57	2.57E+06	11.5	4.47E-04	Not tested		
	85	8.20E+05	1.5	1.83E-04	0	yes	yes
**21**	8	1.31E+06	4	3.05E-04			
	15	2.20E+05	18	8.18E-03	Not tested		
	22	4.70E+05	12.5	2.66E-03	Not tested		
	29	5.48E+06	1.5	2.74E-05	0		
	57	3.15E+06	72	2.29E-03	Not tested		
	85	2.34E+06	2.5	1.07E-04	Not tested	yes	yes

Table S7. Cell pathways for the interrogated mRNA transcripts.

Gene name	Description	Ref.
**DEFA1, Defensin alpha 1**	• family of antimicrobial and cytotoxic peptides involved in host defense and immunomodulatory function,<br>• involved in adaptive (T/B-cells) immunity by serving as chemoattractant and activators of immune cells.	[7]
**SORT1 Sortilin 1**	• functions in protein transport between the Golgi apparatus, endosome, lysosome, and plasma membrane, leading to its involvement in multiple biological processes such as glucose and lipid metabolism as well as and cell death.	[8]
**CCND2 Cyclin D2**	• involved in cell cycle and regulation	[9]
**WNT5A Wingless-type MMTV integration site family, member 5A**	• encodes secreted signaling proteins that have been implicated in oncogenesis and in several developmental processes, including regulation of cell fate and patterning during embryogenesis.	[10]
**FLT3 fms-related tyrosine kinase 3**	• acts as cell-surface receptor for the cytokine FLT3LG and regulates differentiation, proliferation and survival of hematopoietic progenitor cells and of dendritic cells.	[11]
**IL2RA Interleukin 2 receptor, alpha**	• Cytokine-cytokine receptor interaction/ Hematopoietic cell lineage	[12]
**CD19**	B-Lymphocyte Surface Antigen B4, acts as an adaptor protein to recruit cytoplasmic signaling proteins to the membrane and it works within the CD19/CD21 complex to decrease the threshold for B cell receptor signaling pathways.<br>Gene encodes a member of the immunoglobulin gene superfamily. Expression of this cell surface protein is restricted to B cell lymphocytes. This protein is a reliable marker for pre-B cells but its expression diminishes during terminal B cell differentiation in antibody secreting plasma cells. The protein has two N-terminal extracellular Ig-like	[13]

	domains separated by a non-Ig-like domain, a hydrophobic transmembrane domain, and a large C-terminal cytoplasmic domain.

Table S8. Sequences of primers used in gene expression experiments [14].

Gene name, Primer Forward (F) and Reverse (R)	Sequence (5′-3′)	Length (nt)	Tm (°C)	Amplicon size (bp)
DEFA1_F	TCCCTTGCATGGGACGAAAG	20	57.7	111
DEFA1_R	GGTTCCATAGCGACGTTCTCC	21	57.7	
SORT1_F	TGATCTCAGAGGCTCAGTATCC	22	55.4	212
SORT1_R	CAGGACCAATAGCCATGCCAA	21	57.7	
CCND2 _F	CTACCTTCCGCAGTGCTCCTA	21	58.5	163
CCND2 _R	CCCAGCCAAGAAACGGTCC	19	58.9	
JAK2_F	GTATCCACCCAACCATGTCTTC	22	55.4	103
JAK2_R	GTTGCTGCCACTGCAATACC	20	57.1	
WNT5A_F	ATGGCTGGAAGTGCAATGTCT	21	57.2	107
WNT5A_R	ATACCTAGCGACCACCAAGAA	21	55.5	
FLT3_F	TCAAGTGCTGTGCATACAATTCC	23	55.9	148
FLT3_R	AGCAGGGTTAAAACGACAATGA	22	54.8	
IL2RA_F	GGGGACTGCTCACGTTCATC	20	58.1	72
IL2RA_R	GGATCTCTGGCGGGTCATC	19	57.6	
CD19 Ex8-9 _F	TGCCCCGTCTTATGGAAACC	20	57.5	92
CD19 Ex8-9 _R	CTCTTCTTCTGGGCCCACTC	20	57.4	

Table S9. Primer sequences for the detection of alternative splicing in CD19(+) gene from total RNA isolated from cells isolated from B-ALL patients [15].

CD19 **mRNA region (Forward/ Reverse)**	**Sequence (5′-3′)**	**Length (nt)**	**Tm (°C)**	**Amplicon size (bp)**			
				**Full length**	**ΔEx2**	**ΔEx2_part**	**ΔEx5-6**
Exon 1-4 _F	GGAGAGTCTGACCACCATGC	20	57.6	640	374	509	No amplification
Exon 1-4 _R	GGACACAGAGTCAGGGGGTA	20	58.2				
Exon 1-5 _F	GGCCCGAGGAACCTCTA	17	56.2	800	533	669	No amplification
Exon 1-5 _R	CAGCAGCCAGTGCCATAGTA	20	57.2				
Exon 4-8 _F	AAGGGGCCTAAGTCATTGCT	20	56.5	490	No amplification	No amplification	331
Exon 4-8 _R	TGCTCGGGTTTCCATAAGAC	20	54.8				

Table S10. Total RNA mass secured after extraction from cells isolated on anti-CD19 Ab modified chip.

Sample ID	RNA Isolated (ng)
HD3	15.2
HD2	51.2
HD1	14.4
HD4	48.8
p14d15	23.84
p14d29	18.72
p14d57	2.56
p15d8	15.04
p15d15	92.8
p15d29	41.6
p16d8	19.2
p16d15	74.4
p16d22	127.2
p17d8	57.6
p18d8	819.2
MOLT-3, T -ALL cell line	504
SUP-B15 B-ALL cell line	512

References

1. Kruse, A.; Abdel-Azim, N.; Kim, H. N.; Ruan, Y.; Phan, V.; Ogana, H.; Wang, W.; Lee, R.; Gang, E. J.; Khazal, S.; Kim, Y.-M., Minimal Residual Disease Detection in Acute Lymphoblastic Leukemia. *Int J Mol Sci* **2020,** 21, (3), 1054.
2. Alves, G. V.; Fernandes, A. L.; Freire, J. M.; Paiva Ade, S.; Vasconcelos, R. C.; Sales, V. S.; Lemos, T. M.; Gil, E. A.; Freire, F. H.; Cavalcanti e Silva, D. G.; Maciel, J. F.; Farkatt, I. G.; Cavalcanti Júnior, G. B., Flow cytometry immunophenotyping evaluation in acute lymphoblastic leukemia: correlation to factors affecting clinic outcome. *J Clin Lab Anal* **2012,** 26, (6), 431-40.
3. Chantepie, S. P.; Cornet, E.; Salaün, V.; Reman, O., Hematogones: An overview. *Leukemia Research* **2013,** 37, (11), 1404-1411.
4. Kulis, J.; Sędek, Ł.; Słota, Ł.; Perkowski, B.; Szczepański, T., Commonly Assessed Markers in Childhood BCP-ALL Diagnostic Panels and Their Association with Genetic Aberrations and Outcome Prediction. *Genes (Basel)* **2022,** 13, (8).
5. Chiaretti, S.; Zini, G.; Bassan, R., Diagnosis and subclassification of acute lymphoblastic leukemia. *Mediterr J Hematol Infect Dis* **2014,** 6, (1), e2014073.
6. Salzer, W. L.; Burke, M. J.; Devidas, M.; Chen, S.; Gore, L.; Larsen, E. C.; Borowitz, M.; Wood, B.; Heerema, N. A.; Carroll, A. J.; Hilden, J. M.; Loh, M. L.; Raetz, E. A.; Winick, N. J.; Carroll, W. L.; Hunger, S. P., Toxicity associated with intensive postinduction therapy incorporating clofarabine in the very high-risk stratum of patients with newly diagnosed high-risk B-lymphoblastic leukemia: A report from the Children's Oncology Group study AALL1131. *Cancer* **2018,** 124, (6), 1150-1159.
7. te Kronnie, G.; Bicciato, S.; Franceschini, L.; Accordi, B.; Delliorto, M. C.; Rinaldi, A.; Pession, A.; Barisone, E.; Conter, V.; Locatelli, F.; Basso, G., Validation by RQ-PCR and flow cytometry of α-defensin1-3 (DEFA1-3) overexpression in relapsed and refractory acute lymphoblastic leukemia. *Oncol Rep* **2006,** 15, (2), 341-346.
8. Farahi, L.; Ghaemimanesh, F.; Milani, S.; Razavi, S. M.; Akhondi, M. M.; Rabbani, H., Sortilin as a Novel Diagnostic and Therapeutic Biomarker in Chronic Lymphocytic Leukemia. *Avicenna J Med Biotechnol* **2019,** 11, (4), 270-276.
9. Chiles, T. C., Regulation and Function of Cyclin D2 in B Lymphocyte Subsets1. *The Journal of Immunology* **2004,** 173, (5), 2901-2907.
10. Liang, H.; Chen, Q.; Coles, A. H.; Anderson, S. J.; Pihan, G.; Bradley, A.; Gerstein, R.; Jurecic, R.; Jones, S. N., Wnt5a inhibits B cell proliferation and functions as a tumor suppressor in hematopoietic tissue. *Cancer Cell* **2003,** 4, (5), 349-360.
11. Gutierrez-Camino, A.; Richer, C.; Ouimet, M.; Fuchs, C.; Langlois, S.; Khater, F.; Caron, M.; Beaulieu, P.; St-Onge, P.; Bataille, A. R.; Sinnett, D., Characterisation of FLT3 alterations in childhood acute lymphoblastic leukaemia. *British Journal of Cancer* **2024,** 130, (2), 317-326.

12. Carrà, G.; Cartellà, A.; Maffeo, B.; Circosta, P.; Cignetti, A.; Pautasso, M.; Familiari, U.; Volante, M.; Dragani, M. E. E.; Rege Cambrin, G.; Saglio, G.; Morotti, A., Interleukin-2 Receptor Alpha Chain, Also Called CD25, Is a Potential Target in Acute Lymphoblastic Leukemia. *Blood* **2020,** 136, 11-12.
13. Zhou, L. J.; Ord, D. C.; Omori, S. A.; Tedder, T. F., Structure of the genes encoding the CD19 antigen of human and mouse B lymphocytes. *Immunogenetics* **1992,** 35, (2), 102-11.
14. Garza-Veloz, I.; Martinez-Fierro, M. L.; Jaime-Perez, J. C.; Carrillo-Sanchez, K.; Ramos-Del Hoyo, M. G.; Lugo-Trampe, A.; Rojas-Martinez, A.; Gutierrez-Aguirre, C. H.; Gonzalez-Llano, O.; Salazar-Riojas, R.; Hidalgo-Miranda, A.; Gomez-Almaguer, D.; Ortiz-Lopez, R., Identification of differentially expressed genes associated with prognosis of B acute lymphoblastic leukemia. *Dis Markers* **2015,** 2015, 828145.
15. Sotillo, E.; Barrett, D. M.; Black, K. L.; Bagashev, A.; Oldridge, D.; Wu, G.; Sussman, R.; Lanauze, C.; Ruella, M.; Gazzara, M. R.; Martinez, N. M.; Harrington, C. T.; Chung, E. Y.; Perazzelli, J.; Hofmann, T. J.; Maude, S. L.; Raman, P.; Barrera, A.; Gill, S.; Lacey, S. F.; Melenhorst, J. J.; Allman, D.; Jacoby, E.; Fry, T.; Mackall, C.; Barash, Y.; Lynch, K. W.; Maris, J. M.; Grupp, S. A.; Thomas-Tikhonenko, A., Convergence of Acquired Mutations and Alternative Splicing of CD19 Enables Resistance to CART-19 Immunotherapy. *Cancer Discov* **2015,** 5, (12), 1282-95.

Disclaimer/Publisher's Note: The statements, opinions and data contained in all publications are solely those of the individual author(s) and contributor(s) and not of MDPI and/or the editor(s). MDPI and/or the editor(s) disclaim responsibility for any injury to people or property resulting from any ideas, methods, instructions or products referred to in the content.
